# Identification of novel diabetes impaired miRNA-transcription factor co-regulatory networks in bone marrow-derived Lin^-^/VEGF-R2^+^ endothelial progenitor cells

**DOI:** 10.1371/journal.pone.0200194

**Published:** 2018-07-11

**Authors:** Mohammad R. Irhimeh, Mohamed Hamed, Daniel Barthelmes, Yvonne Gladbach, Volkhard Helms, Weiyong Shen, Mark C. Gillies

**Affiliations:** 1 Cell and Tissue Therapies WA, Royal Perth Hospital; Medical Unit, Fiona Stanley Fremantle Hospitals Group, Perth, WA Australia; 2 Save Sight Institute, Sydney Hospital and Sydney Eye Hospital, Central Clinical School, The University of Sydney, Sydney, NSW Australia; 3 Institute for Biostatistics and Informatics in Medicine and Ageing Research, Rostock University Medical Centre, Rostock University, Rostock, Germany; 4 Center for Bioinformatics, Saarland University, Saarbrücken, Germany; Centro Cardiologico Monzino, ITALY

## Abstract

Endothelial progenitor cells (EPCs) are a group of rare cells that play an important role in the repair of injured vascular endothelial cells and assist in reperfusion of ischemic tissue. Decreased production and/or loss of function of EPCs are associated with diabetic vasculopathy. The molecular mechanisms by which diabetes impairs EPCs remain unclear. We conducted microarray experiments followed by integrative regulatory analysis on cells isolated from Akita diabetic mice (18-weeks after onset of diabetes) and age-matched non-diabetic controls. Two types of cells were isolated from mice bone marrow; Lin^+^ cells and Lin^-^/VEGF-R2^+^ EPCs. RNA was hybridized to *mouse WG-6 V2 beadchips* followed by comprehensive gene network analysis and computational validation of the obtained results. In total, 80 genes were exclusively DE between non-diabetic Lin^-^/VEGF-R2^+^ EPCs and diabetic Lin^-^/VEGF-R2^+^ EPCs, of which the 3 genes *Clcnka*, *Pik3c2a*, and *Ptf1a* are known to be associated with diabetic complications. Further analysis led to the establishment of a TF-miRNA mediated regulatory network specific to diabetic Lin^-^/VEGF-R2^+^ EPCs and to identify 11 central-hub TFs (*Tbp*, *Ahr*, *Trp53*, *Gata1*, *Foxo1*, *Foxo4*, *Yy1*, *Max*, *Pparg*, *Myc*, *Cebpa*), and 2 miRNAs (*mir-139-5p*, *mir-709*) that might act as putative genomic drivers of diabetic pathogenesis in Lin^-^/VEGF-R2^+^ EPCs. Moreover, we identified multiple TF-miRNA co-regulatory network motifs for which we validated their contribution to diabetic Lin^-^/VEGF-R2^+^ EPCs in terms of statistical significance and relevance to biological evidence. Our findings suggest that diabetic Lin^-^/VEGF-R2^+^ EPCs have specifically altered signature genes and miRNAs that render their capacity to proliferate and differentiate.

## Introduction

Chronic diabetes is associated with injury of vascular endothelial cells (ECs) [1] that is believed to be repaired by neighboring resident endothelial cells, resident endothelial progenitor cells (EPCs), and bone marrow (BM) derived EPCs [2]. It has been reported that diabetes is associated with impairment of EPC function [3]. Diabetic patients were shown to have reduced EPC numbers in the peripheral blood (PB) [4] and the isolated PB EPCs showed impaired proliferate, tubes formation, adhesion and were less effective in repairing vascular injuries [5]. Several studies suggest that reduced number and/or dysfunction of EPCs in cell mobilization, proliferation, adhesion, and incorporation into the vasculature may contribute to diabetic vascular complications [6].

Recently, we reported an impaired mobilization capacity of BM Lin^-^/VEGF-R2^+^ EPCs in diabetic mice [7]. EPCs are usually defined based on their surface markers and proliferative and clonogenic potential and they are believed to be lineage and functionally heterogeneous [2]. It has been suggested that an insult to the stem cell niche might contribute to reduction in the numbers and impairment of EPC function [8]. These Lin^-^/VEGF-R2^+^ EPCs play an important role in regenerating the endothelium through migration, proliferation, differentiation and by secreting pro-angiogenic cytokines [9].

The majority of molecular studies on the impairment of diabetic EPCs (D-EPCs) function have been conducted on human EPCs isolated from PB after long history of diabetes. Thus, little is known about the changes occurring in BM in the early stages of diabetes. Therefore, we intended to investigate the effect of diabetes on BM Lin^-^/VEGF-R2^+^ EPCs molecular signature using isolated cells from Akita diabetic mice and age-matched non-diabetic controls. A microarray analysis and gene differential expression (DE) combined with our newly established analytical methods including TFmiR [10, 11] were used to identify regulatory networks that potentially drives the diabetic vasculopathy. Unique 3-node Feed Forward Loop (FFL) co-regulatory motifs consisting of a Transcription factors, miRNA, and co-targeted genes were identified and validated statistically and biologically.

## Research design and methods

### Animals and Lin^-^/VEGF-R2^+^ EPCs isolation and characterization

Ethics approval was obtained from University of Sydney Animal Ethics Committee. Ins2^Akita^ mice carry a dominant point mutation in the Insulin-2 gene on chromosome-7 resulting in the development of diabetes at four weeks after birth. As females develop diabetes more slowly and less stably compared with males, only males heterozygous for Ins2^Akita^ allele (diabetic) and males homozygous for wildtype Ins2 allele (non-diabetic) were used. Once diabetes was established (blood glucose level>13.3mmol/L), mice were monitored weekly for changes in bodyweight and blood glucose levels for 18 weeks. BM Lin^-^/VEGF-R2^+^ EPCs from diabetic and age matched controls were collected as we described before [2, 7]. BM cells were Fc-blocked, and then stained with APC-mouse-lineage-antibody-cocktail and FITC-rat-anti-mouse-VEGF-R2. After that, cells were washed and incubated with APC-magnetic-particles-DM. Hematopoietic lineage markers (CD3e/CD11b/CD45R/Ly-76/Ly-6G/Ly-6C) were magnetically depleted producing Lin^-^ and Lin^+^ fractions. The Lin^-^ fraction was further enrichment magnetically using anti-FITC VEGF-R2 beads producing Lin^-^/VEGF-R2^+^ EPCs. Phenotypic properties of the isolated Lin^-^/VEGF-R2^+^ EPCs were characterized using MACSQuant Analyzer-10 flow cytometerand were tested for cobble-stone colony formation, developing tube like structures on Matrigel, Dil-ac-LDL uptake and UEA-I staining as previously described [2, 7].

### Group design and comparisons

**Fig 1:**
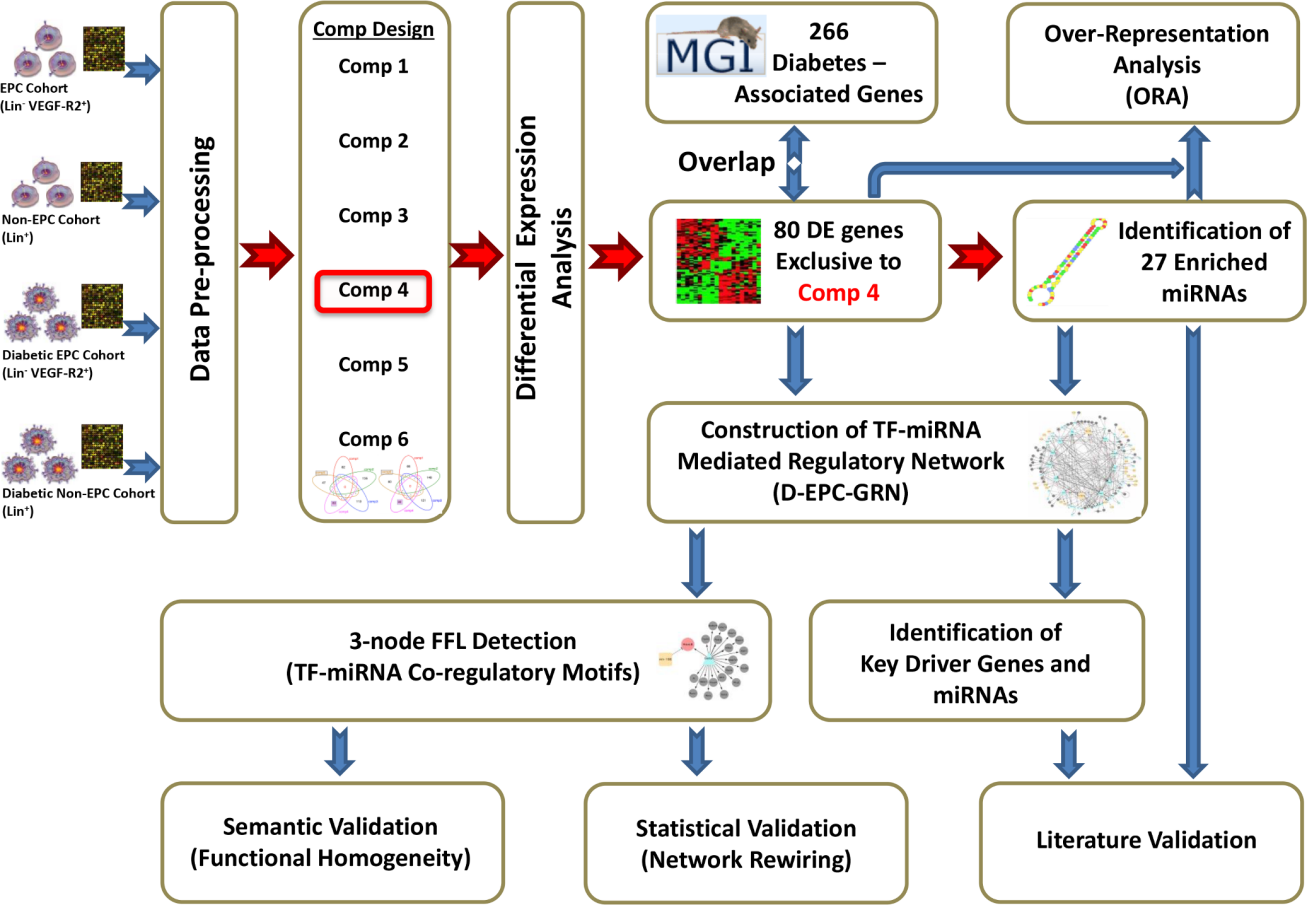
Schematic diagram showing the study experimental design, the integrative computational analysis, and the validation procedures used in this study. See Fig 2 for the list of comparisons conducted between the pairs of samples.

Four experimental groups were established: non-diabetic non-EPCs (Lin^+^) cells, diabetic non-EPCs (Lin^+^) cells, non-diabetic EPCs (Lin^-^/VEGF-R2^+^) and diabetic EPCs (Lin^-^/VEGF-R2^+^). The Lin^+^ cells were used as an internal reference to identify DE occurring not exclusively in Lin^-^/VEGF-R2^+^ cells. Six different comparisons were conducted between the four groups (Fig 1) allowing us to distinguish DE which specifically occurred in Lin^-^/VEGF-R2^+^ D-EPCs from that occurring in other phenotypes of hematopoietic lineage committed BM cells. Hence, only significant changes in gene expression observed in diabetic vs non-diabetic Lin^-^/VEGF-R2^+^ EPCs that did not occur in the Lin^+^ population were considered in the final analysis.

### RNA isolation and array processing

RNA isolation was performed using Qiagen RNeasy-Mini Kit. RNA concentration and integrity were assessed using the BioRad Experion automated electrophoresis system on a RNA StdSens Chip (BioRad, 700–7159). Later, total-RNA was quality and quantity ascertained using the Agilent Bioanalyser2100 using NanoChip protocol. A total of 500ng was labelled using the Ambion Total Prep RNA amplification kit (IL1791). A total of 1.5μg of labelled cRNA was then prepared for hybridization to the *Mouse WG-6 V2 beadchip* by preparing a probe cocktail (cRNA at 0.05μg/μl) that includes GEX-HYB Hybridization Buffer. A total hybridization volume of 30μl was prepared for each sample and loaded into a single array on the *beadchip*, which was hybridized as outlined in the Illumina manual protocols. Chips were then coupled with Cy3 and scanned in the Illumina iScan scanner and its software, GenomeStudio, converted the signal on the array into a TXT file for analysis.

### Data processing

The research aim is to unveil the molecular mechanisms and mutual interactions between deregulated coding and non-coding RNAs that underlie the early stages of diabetes in BM Lin^-^/VEGF-R2^+^ EPCs. Therefore, it was sufficient to measure the expression at single time point, but only after ensuring developing the corresponding phenotype. Raw expression values were background corrected, log_2_ transformed and quantile normalized using the lumiR package [12] of the Bioconductor suite [13]. Expression profiles of redundant probe sets were merged by computing the mean of all probes related to single genes as reported previously [11]. A list of 266 diabetic-associated genes was downloaded from the Mouse Genome Informatics (MGI) database [14].

### Differential expression analysis

The six comparisons were compared by DE analysis using three methods each: significance analysis of microarray (SAM) [15], moderated t-test [16], and area-under-the-curve of the receiver operator characteristics (AUC ROC) [16]. Genes that were classified as DE by at least two of those three methods were included in the list of DE-genes. We focused on genes that are exclusively involved in the fourth comparison only. Expression heat-maps and PCA-plots were generated by R [17].

### miRNA enrichment

We predicted the miRNAs associated with the list of DE-genes by determining the set of miRNAs whose target genes and regulator TFs are significantly enriched within the DE gene-set using the hypergeometric distribution function followed by the Benjamini-Hochberg adjustment with a cutoff value of 0.01. The miRNA target-genes and the miRNA regulators were compiled from the regulatory databases listed in [10].

### Construction of TF-miRNA regulatory networks and motif modules

The regulatory interactions between the DE-genes and their targets/regulators as well as the interactions between the enriched miRNAs and their regulators/targets were retrieved from the databases of TFmiR webserver [10]. Only regulatory interactions that are supported by experimental evidence were included in this analysis. To identify central-hubs (key network-players), we computed the degree centrality measure for the entire network using R igraph-package [18] and highlighted the top 10% highest centrality nodes of the TFs/genes and miRNAs. Next, 3-node FFL-motifs (recurring network patterns consisting of a miRNA, a TF, and a joint target gene) were characterized using the computational procedure described in [10]. The networks and co-regulatory motifs were visualized with Cytoscape-V3.3.0 [19].

### Network validation and assessment of key player-nodes

#### 1- Statistical validation: Significance of the detected motifs

To evaluate the significance of each co-regulatory motif, we compared how often it occurs in the full regulatory network against the number of times it is detected in ensembles of randomized networks preserving the same node degrees. The randomization procedure is explained in detail in [10]. The random networks were constructed 100-times and compared to the real network. The p-value was calculated as

$$p-value=\frac{N_{h}}{N_{r}}$$

where Nh is the number of random times that a certain motif type is detected more than or equal to its number in the real network, and Nr is 100. Only motifs having p-value<0.05 were considered for further analysis.

#### 2- Semantic validation: Functional homogeneity within the motif nodes

In order to assess the biological relevance of the identified network motifs and to better understand their functional roles, we analyzed the Gene Ontology (GO) semantic similarity for all pairs of genes regulated by the TF or the miRNA of each motif. The GoSemSim R-package [20] was used to calculate the semantic similarity scores according to the GO annotations. Statistical significance was determined by randomly selecting the same number of co-regulated genes (genes targeted by either the TFs or the miRNA) from all Entrez genes with GO annotations, and computing their similarity scores. The permutation procedure was repeated 100-times. Then, Kolmogorov-Smirnov test was utilized to check whether the functional similarity scores of all gene-pairs composing a regulatory motif are significantly higher than that of randomly selected pairs.

#### 3- Over-representation analysis for genes and miRNAs

The functional enrichment analysis was conducted as we reported previously [21]. Briefly, enriched KEGG Pathways and GO functional categories were identified using the DAVID tool [22]. For this, we analyzed which pathway/functional terms were annotated to at least 2 genes and were statistically over-represented in the study gene-set against the full mouse genome (control). Enrichment was evaluated through the hyper-geometric test (p-value≤0.05). Enrichment analysis of the miRNAs sets was performed using the TAM tool [23].

## Results

We developed and applied an integrative approach to conduct combinatorial regulatory network analysis in the context of early diabetes in Lin^-^/VEGF-R2^+^ EPCs with the aim of identifying the major genetic drivers and the essential network modules that could possibly dissect how diabetes impairs EPC functions. Fig 1 illustrates the implemented integrative approach including the study experimental design, the downstream analysis, and the applied validation steps.

### Establishing diabetes in mice

By 22 weeks of age (time of BM collection) the mean bodyweights for non-diabetic and diabetic mice were 37.2±3.1g and 23.0±2.2g, respectively (p<0.0001). The mean blood glucose levels of non-diabetic and diabetic mice were 9.3±1.4[7.5–10.0]mmol/L and 33.1±2.9[27.5–33.4]mmol/L, respectively (p<0.0001). The mean HbA1c was higher in the Akita group (11.5±0.5%) compared to the control (4.6±0.3%).

### Cell numbers, characteristics, function and RNA quality and quantity

The mean percentages of Lin^+^ and Lin^−^/VEGF-R2^+^ cells in BM nucleated cells fraction were 80.7±4.7% and 4.5±2.1%, respectively. After adjusting for bodyweight, the number of Lin^−^/VEGF-R2^+^ EPCs in diabetic BM 5.6[4.0–9.3]×10^4^ /g bodyweight was slightly higher than non-diabetic 5.0[2.9–7.2]×10^4^ /g bodyweight.

Akita non-diabetic and diabetic Lin^-^/VEGF-R2^+^ cells demonstrated the inherent and essential properties of EPCs such as cobble-stone colony formation, ability to be cultured and passaged, phenotypic analysis on flow cytometry, developing tube like structures on Matrigel, Dil-acLDL uptake and staining for UEA-I.

Akita mice develop diabetes spontaneously, as a result of a progressive loss of beta-cell function and decreased pancreatic beta-cell density, mimics DM type 1 but do not show late stage complications. This model demonstrated that early diabetic changes can be detected in Lin^-^/VEGF-R2^+^ EPCs function such as lose of mobilize potential from BM to PB and the reduction in their capability to migrate towards injured blood vessels and repair mechanism.

Only samples that had a high RNA yield and RQI≥7 (mean was 8.9) were selected for microarray analysis. For the non-diabetic and diabetic Lin^+^ fraction, the concentrations of extracted RNAs were 202.7±122.0ng/μl and 283.0±114.7ng/μl, respectively (p = 0.108). The Lin^-^/VEGF-R2^+^ EPCs fraction yielded 97.2±80.0ng/μl RNA from non-diabetics and 157.3±87.6ng/μl RNA from diabetics (p = 0.198). Before microarray processing, the RNA integrity was measured again and the RNA integrity number was ≥7 (mean 8.6) in all samples.

### Differential expression analysis

**Fig 2:**
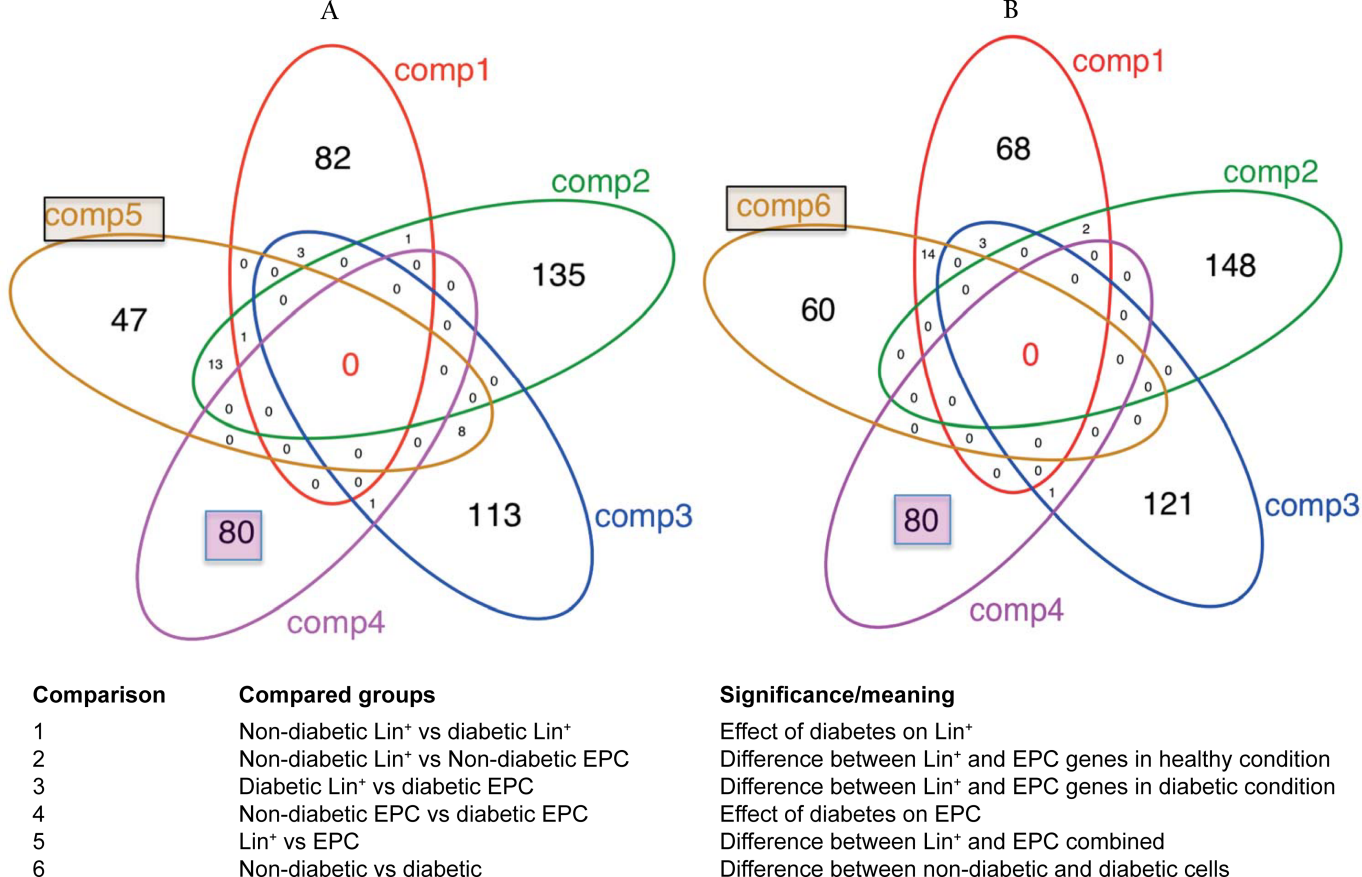
Venn diagrams showing overlapping differentially expressed genes among the six comparisons. (A) Comparisons 1–5, (B) comparisons 1–4 and 6. In both Venn diagrams the same 80 genes were found specific to comparison 4 (non-diabetic EPCs vs D-EPCs).

**Fig 3:**
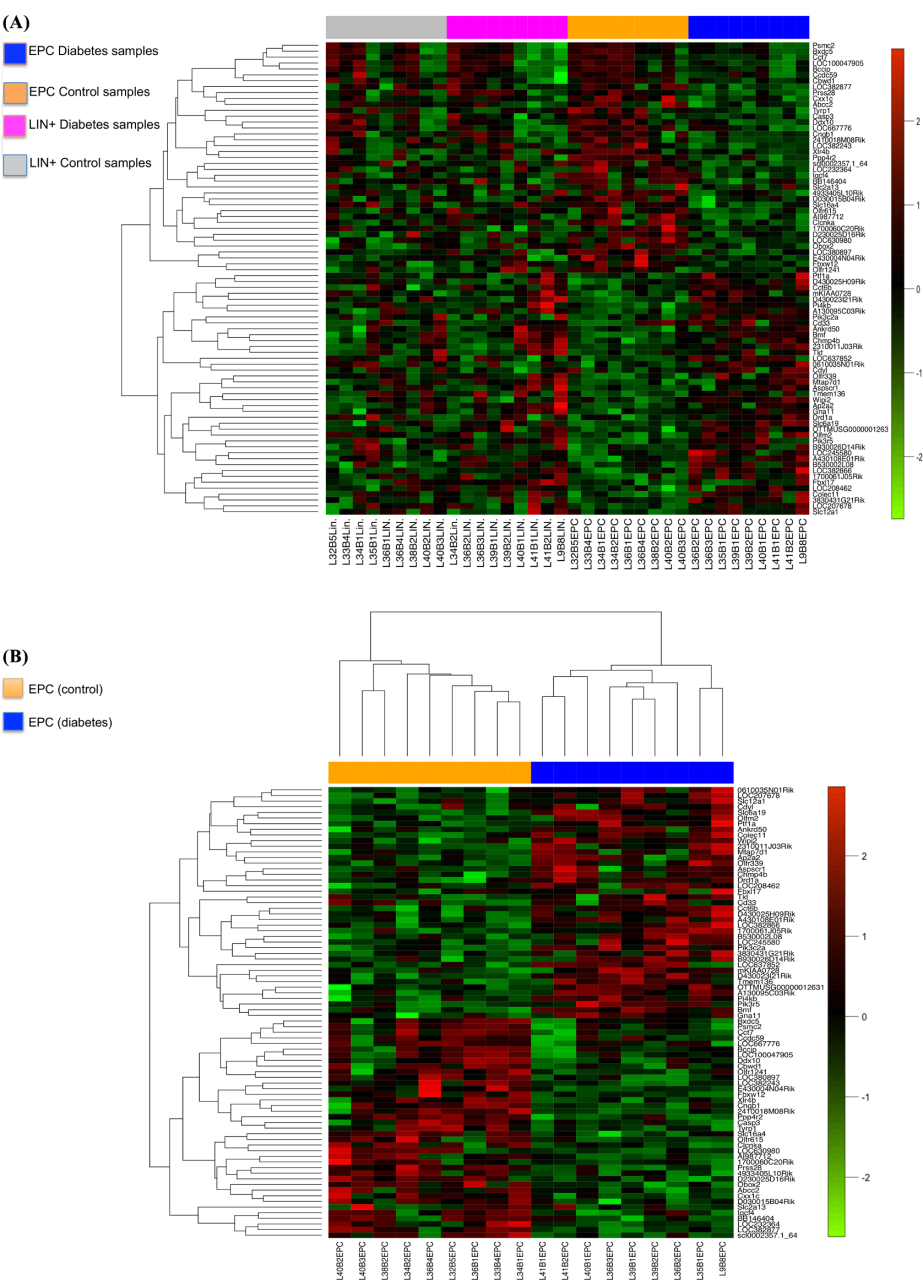
Heat maps of the microarray analysis results. **(A):** Differentially expressed genes in all 36 samples. Green-spots represent down-regulated genes, and red-spots represent up-regulated genes. The blue color represents D-EPCs, the orange color represents the non-diabetic EPCs, the blue pink represents diabetic Lin^+^, and the gray color represents the non-diabetic Lin^+^. **(B):** 80 core enrichment genes in comparison-4 (non-diabetic vs D-EPCs). Green spots represent down-regulated genes, and red spots represent up-regulated genes. The order of genes is obtained by hierarchical clustering. The orange color represents the non-diabetic EPCs while the blue color represents the D-EPCs.

Comparing non-diabetic Lin^-^/VEGF-R2^+^ EPCs with Lin^-^/VEGF-R2^+^ D-EPCs identified 80 DE-genes that were exclusively detected in the fourth comparison as shown in the Venn diagrams (Fig 2). The 80 DE-genes were significantly associated with the known ‘diabetes-associated genes’ list (hyper-geometric test, p = 0.0319), see methods section. Out of the 80 DE-genes (S1 Table), three genes *Clcnka* (downregulated) and *Ptf1a* and *Pik3c2a* (both up-regulated) are shared with the diabetes-associated genes list. A heat-map was generated to show the relative gene expression among the four groups (Fig 3A). Then non-diabetic and Lin^-^/VEGF-R2^+^ D-EPCs were selected to generate a heat-map for the relative expression of the 80 DE-genes (Fig 3B). To show how the 80 DE-genes are separated between non-diabetic and Lin^-^/VEGF-R2^+^ D-EPCs, PCA analysis was conducted (S1 Fig). The PCA clustered the DE-genes into down-regulated and up-regulated genes based on their relative expression levels.

### miRNA enrichment analysis

27 miRNAs were identified by determining the set of miRNAs whose target genes and regulator TFs were significantly enriched within the 80 DE-genes. The identified miRNAs are: *mir-1*, *mir-133a*, *mir-200*, *mir-429*, *mir-141*, *mir-451*, *mir-709*, *mir-103-1*, *mir-148b*, *mir-182*, *mir-96*, *mir-183*, *mir-205*, *mir-378*, *mir-146a*, *mir-124*, *mir-210-3p*, *let-7c*, *mir-139-5p*, *mir-124a*, *mir-223*, *mir-145*, *mir-196*, *mir-200a*, *mir-200b*, *mir-29c*, *mir-27b*. The biological role of these miRNAs was assessed by linking them to functional and disease annotations via Over Representation Analysis, S2 Table. Interestingly, the identified miRNAs were significantly enriched with EPC-related biological processes such as diabetes mellitus (p< 0.013), cell proliferation (p-value 0.0008), cell differentiation (p-value 0.007), cell fate determination (p<0.03), hematopoiesis (p<0.035), glucose metabolism (p-value <0.029), and carbohydrate metabolism (p-value 0.0001).

### Construction of TF-miRNA mediated regulatory network

Next, we constructed a gene regulatory network (GRN) that represents the combinatorial regulatory interactions between the DE-genes in Lin^-^/VEGF-R2^+^ D-EPCs and the enriched miRNAs. The GRN contains three types of nodes, namely miRNAs, TFs (nodes regulating genes and miRNAs), and target genes (nodes regulated by TFs or miRNAs), S2 Fig. We refer to this GRN-network as “D-EPC-GRN”. To evaluate the contribution of each node-type in the D-EPC-GRN stability and robustness, the number of connections that each node is involved in (also termed node-degree centrality) were computed and ranked according to this number. The most connected nodes (miRNAs, TFs, and target genes) were candidates for central-hubs that could possibly drive the diabetes etiology in Lin^-^/VEGF-R2^+^ EPCs and thus could act as potential master-regulators. Eleven TF/gene hubs were identified (*Myc*, *Pparg*, *Gata1*, *Max*, *Cebpa*, *Ahr*, *Tbp*, *Trp53*, *Foxo1*, *Foxo4*, *and Yy1*) and two miRNA hubs (*mir-709*, *and mir-139-5p*) (S2 Fig). Among them, *Foxo1*, *Myc*, *Yy1*, *and Cebpa* were reported in literature to be essential TFs in regulating diabetes [24–27].

### Identification of TF-miRNA co-regulatory motifs

Biological networks often contain recurring interconnection patterns known as FFL motifs [28] that control different aspects of cell functions and diseases [29, 30]. Therefore, the presence of 3-node co-regulatory FFL-motifs comprising the DE TFs/genes and the miRNAs in the D-EPC-GRN was checked. This led to the detection of 55 significant co-regulatory motifs in the constructed D-EPC-GRN (S3 Table). The statistical significance of the motifs was tested by comparing their count in the real network to their counts in randomized variants of these networks preserving the same node degrees.

**Fig 4:**
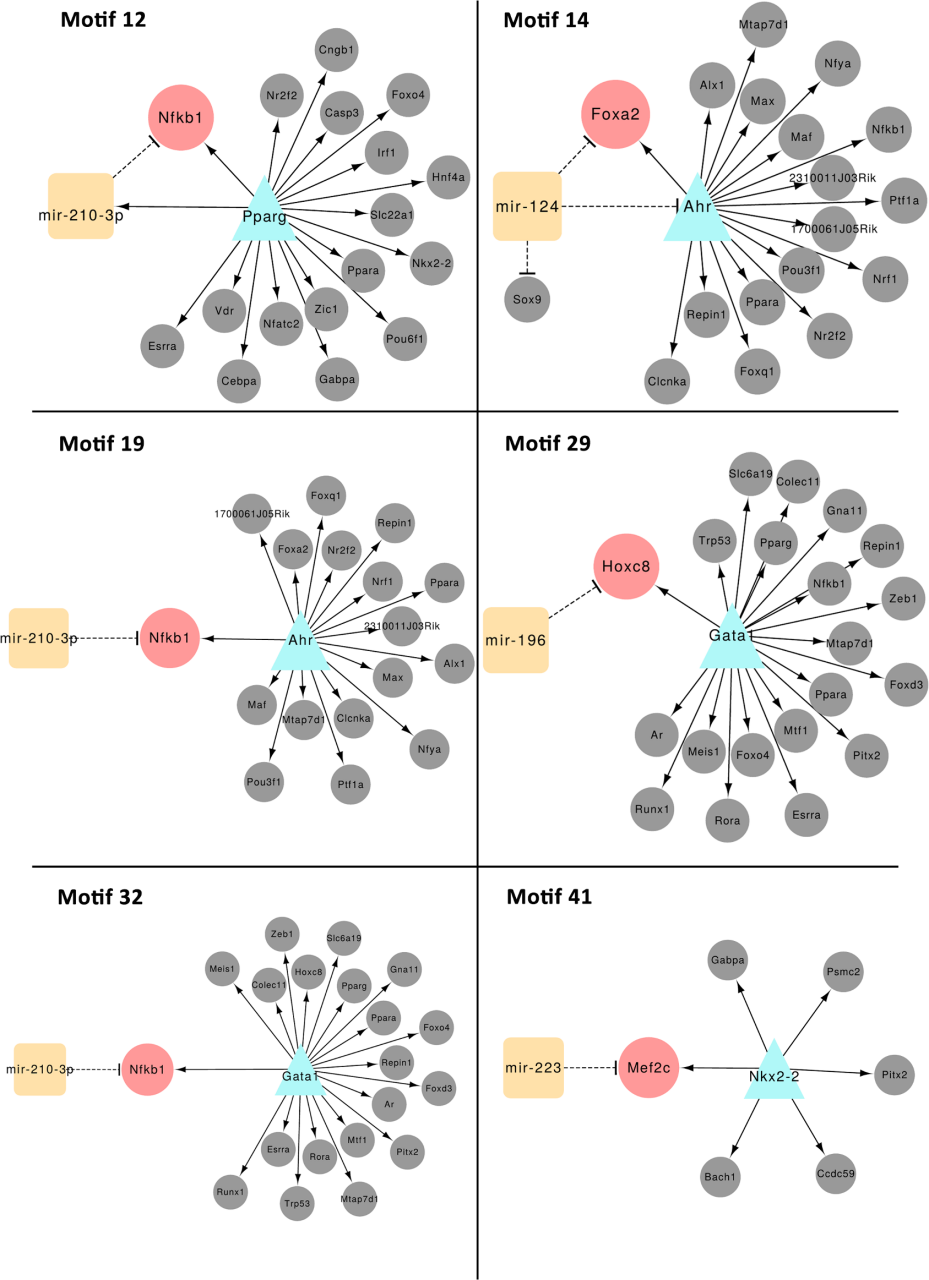
Six interesting co-regulatory motifs suggesting a cooperative functional role between the identified central-hubs and their potential partners of miRNAs, TFs, and target genes in the D-EPC-GRN network. TFs are represented by a turquoise triangle whereas miRNAs are shown as orange squares. Pink nodes denote the common target genes and the grey nodes depict the co-regulated genes.

Fig 4 illustrates six interesting motifs detected in the D-EPC-GRN that appear to have strong relevance to the regulation of murine diabetes. Each of the motifs contains a specific EPC central gene and/or central miRNA that are regulating a deregulated set of target genes including two of the three identified diabetes-associated genes (*Clcnck* and *Ptf1a*) in motifs 14 and 19.

**Fig 5:**
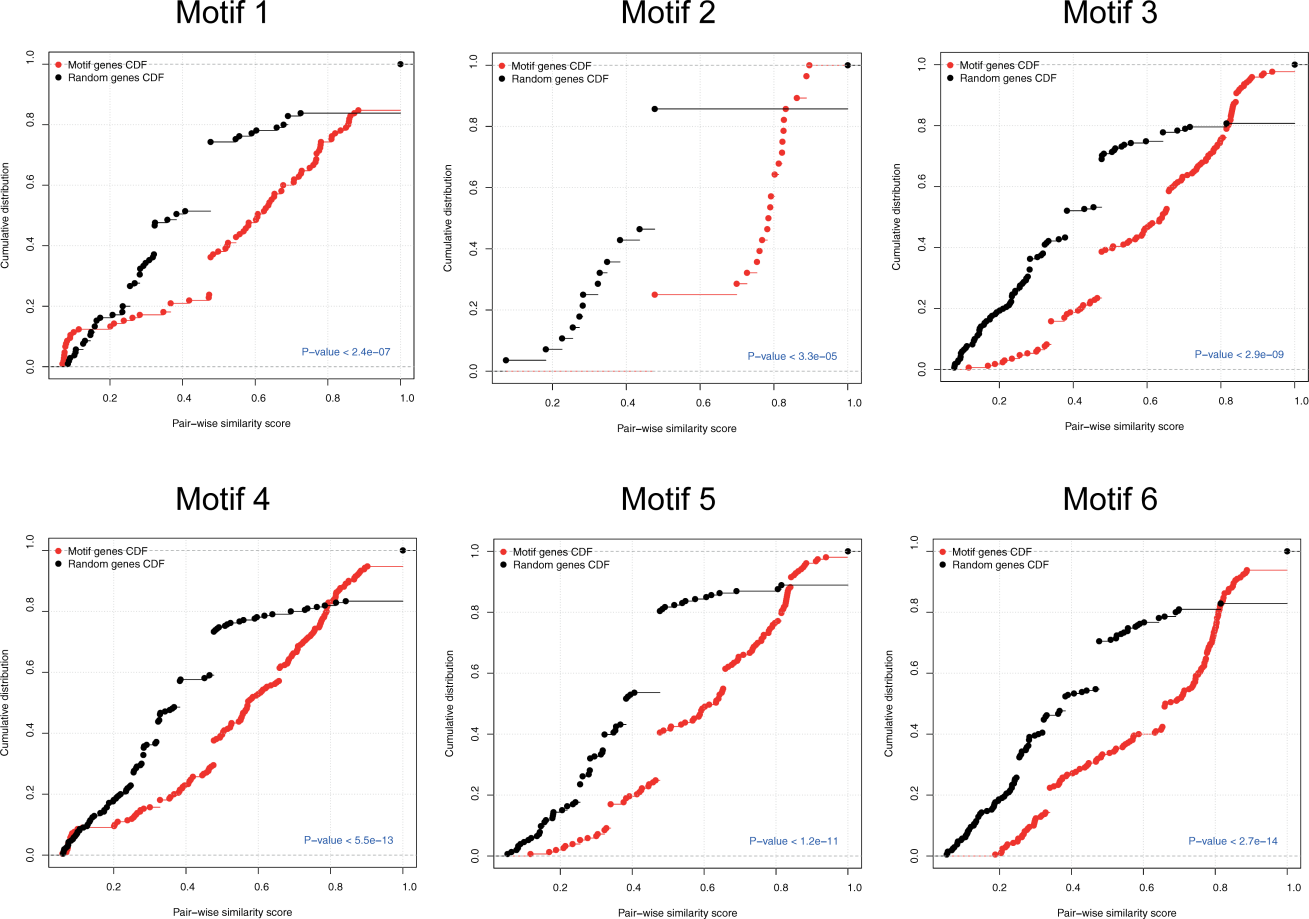
Functional homogeneity of the visualized six motifs. Cumulative distribution of GO functional semantic scores of gene pairs of co-regulated genes in the examined six motifs (red) versus randomly selected genes (black). The p-value was calculated using the Kolmogorov-Smirnov test.

Next, we assessed the biological evidence of these motifs to better understand their functional roles in driving the diabetes in Lin^-^/VEGF-R2^+^ EPCs. To this end, we calculated the functional similarity scores between co-regulated gene-pairs as a measure of their functional homogeneity and integrity. The distribution of the resulting similarity scores was compared to the similarity score distribution of randomly selected gene-pairs (p<8.8e^-5^, Kolmogorov-Smirnov test), Fig 5. The results show that the co-regulated genes have significantly more similar cellular functions than the randomly selected genes. This indicates that these six FFL-motifs could potentially hint at dissecting the related dysfunctions and molecular changes in Lin^-^/VEGF-R2^+^ EPCs during early diabetes.

## Discussion

The direct effect of high glucose on EPCs function is still controversial with most studies being done on human EPCs. As is the case for research into surface markers of EPCs, in which conflicting results have sometimes been presented, the field of diabetes induced changes in EPCs is replete with paradoxical or even contradictory results. One factor contributing to the confusion is the fact that the condition “diabetes” when used in experiments or studies is ill-defined. It comprises patients with quite different stages of the disease and different complications. Another critical point naturally is the definition of EPCs, which also varies from study to study. Despite a certain degree of confusion, the mechanisms of diabetes related changes become clearer as more and more studies examining EPCs in diabetes from different angles become available. One of the most significant findings in Akita model come from the analysis of gene-expression data of genes known to be involved in Lin^-^/VEGF-R2^+^ function and mobilization. This showed that early diabetes leads to significant changes in Lin^-^/VEGF-R2^+^ EPCs function.

The effect of diabetes on Lin^-^/VEGF-R2^+^ EPCs number and function has previously been investigated. Although EPC-types and the methods used were quite different they all returned significant dysfunction of Lin^-^/VEGF-R2^+^ D-EPCs. We previously demonstrated that Lin^-^/VEGF-R2^+^ EPCs defined as Lin^−^/VEGF-R2^+^ were more primitive than other Lin^-^/VEGF-R2^+^ EPCs described in literature and due to diabetes they had limited vascular repair capacity attributed to their impaired ability to mobilize, rather than their ability to proliferate, leading to Lin^-^/VEGF-R2^+^ EPCs BM trapping [2, 7, 31]. Since the exact mechanism underlying this impaired mobilization is still unknown, identifying the responsible genes, TFs, miRNAs, may lead to valuable information. Numerous explanations for Lin^-^/VEGF-R2^+^ D-EPCs dysfunction have been proposed, including increased oxidative stress, NADPH oxidase activation, altered nitric oxide pathway and increased inflammatory cytokines [32]. However, and since diabetes is a complex pathophysiological syndrome, it is unlikely that Lin^-^/VEGF-R2^+^ D-EPCs dysfunction could be explained by a single independent mechanism. Hence, this study used microarray, well-established integrative data analysis, and multiple validation procedures [21] to identify genes, TFs and miRNAs in Lin^-^/VEGF-R2^+^ D-EPCs and could potentially lead to their dysfunction. Consequently, we identified a novel TF-miRNA GRN specific to Lin^-^/VEGF-R2^+^ D-EPCs (D-EPC-GRN), putative genomic drivers (TFs and miRNAs) and significant FFL network modules that involve dysregulated TFs/genes, associated miRNAs as well as diabetes-associated genes. In the Lin^-^/VEGF-R2^+^ D-EPC-GRN, 13 central-hubs were identified (11 genes: *Pparg*, *Foxo1*, *Foxo4*, *Ahr*, *Trp53*, *Gata1*, *Yy1*, *Max*, *Myc*, *Cebpa*, *Tbp*, and 2 miRNAs: *mir-139-5p*, *mir-709*) as potential master-regulatory genes/miRNAs or essential genetic-drivers of the molecular pathology of Lin^-^/VEGF-R2^+^ D-EPCs.

*Ppara* and *Pparg* genes that are associated with type-2 diabetes, hepatic metabolic response to diabetes, insulin resistance through their expression in vasculature cells and EPC trafficking regulation [33], were significantly upregulated. Activation and over-expression of *Ppara* suppresses EPCs mobilization and hypoxia induced homing, which occur through the inhibition of the *HIF-1α/SDF-1* pathway [34]. In this study, *Ppara* upregulation in Lin^-^/VEGF-R2^+^ D-EPC-GRN was regulated mainly by *Pparg* and *Ahr* central-hubs and was consistent with an anti-angiogenic role and impaired *HIF-1α/SDF-1-Ppara* axis.

**Fig 6:**
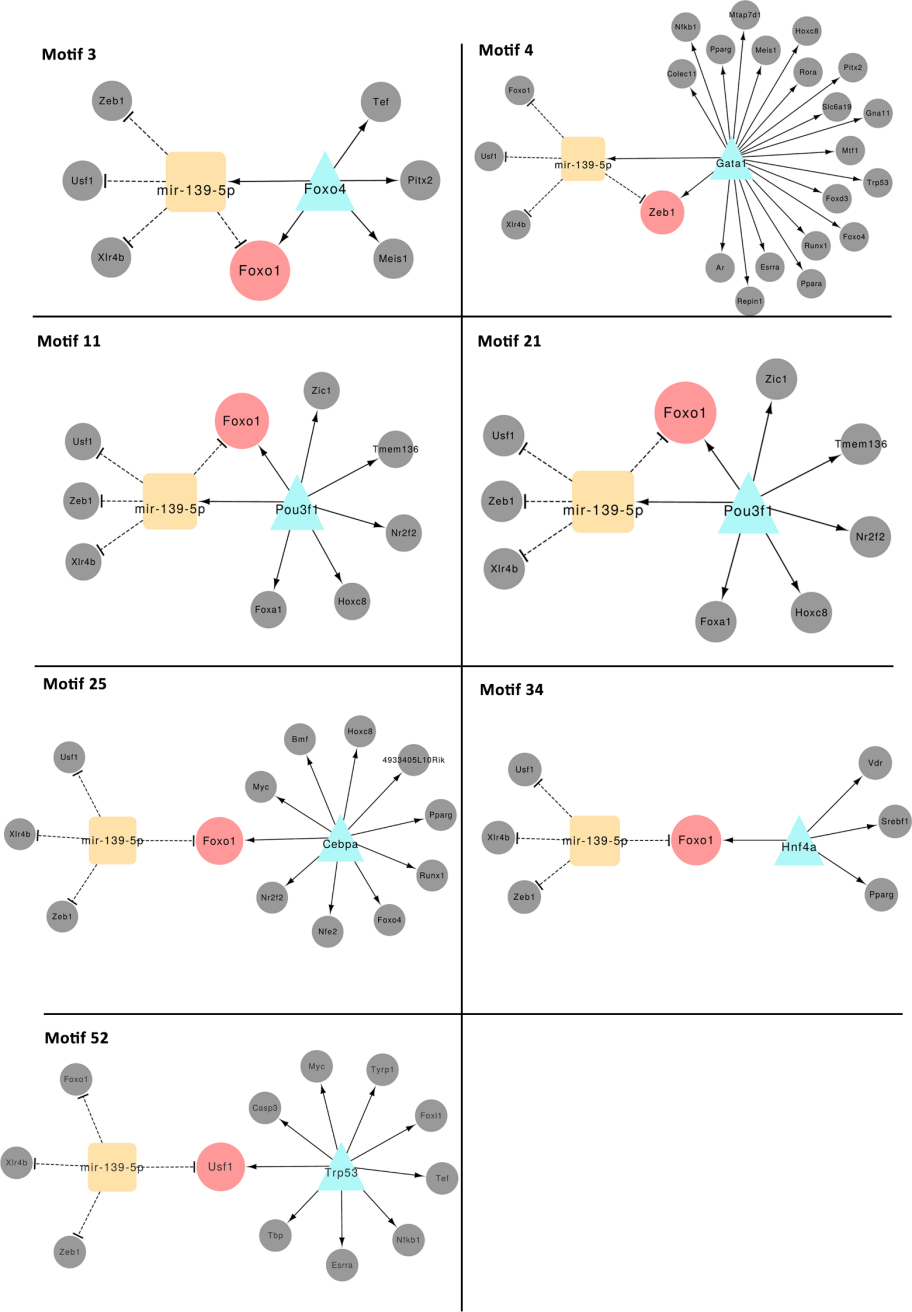
Visualization of all motifs that include mir-139-5p and its interactions with central-hubs.

*Foxo1 and Foxo4* upregulation was implicated in diabetes, diabetic complications and cardiovascular disease, through impairing proliferation and differentiation, and abnormal cytokine expression, inflammation, and resistance to oxidative stress [35]. Two of the major central-hubs in Lin^-^/VEGF-R2^+^ D-EPC-GRN were *Foxo1* and *Foxo4* and *mir-139-5p* was identified as their key regulator, which also regulates other key diabetic TFs (*Zeb1*, *Usf1*, and *Xlr4b*) (Fig 6).

We also found that *Gata1* regulates *mir-139-5p* and *Zeb1*, while *Pou3f1* regulates *mir-139-5p* and *Foxo1*. At the same time, *mir-139-5p* suppresses *Zeb1*, *Foxo1*, *Usf1* and *Xlr4b* (Supplementary material motifs 4 and 11). This led to the identification of an important diabetic regulatory mechanism in impaired Lin^-^/VEGF-R2^+^ EPCs. The activity of *Foxo* family is tightly regulated by post-translational modification, including phosphorylation, acetylation, and ubiquitylation. *Foxo1* plays a crucial role in regulating gluconeogenesis and glycogenolysis by insulin signaling [36]. Another diabetic DE-gene in the D-EPC-GRN is *Pik3c2a*, which encodes for the Pik3c2a enzyme that is activated by insulin. Thus, in diabetes Pik3c2a enzyme activity is expected to be suppressed, which may result in up-regulation of *Foxo1*. In our study, both *Pik3c2a* and *Ptf1a* (annotated as diabetic-specific genes in the MGI database) were differentially up-regulated in the D-EPC-GRN. *Pik3c2a* as well as *Foxo4* were regulated by *Ikzf1* (linked to type-1 diabetes [37]) whereas *Ptf1a* was regulated by the *Ahr*. This suggests essential regulatory roles of Lin^-^/VEGF-R2^+^ EPCs’ *Foxo4* and *Ahr* in the early onset of diabetes.

The other mechanism by which *Foxo* may regulate Lin^-^/VEGF-R2^+^ D-EPCs is via the oxidative stress activated *P66shc-Akt-Foxo* pathway [38]. *P66shc* was reported to be involved in EPC dysfunction due to hyperglycemia [39]. In this study, *Foxo1&4* were DE in Lin^-^/VEGF-R2^+^ D-EPCs, which may contribute to Lin^-^/VEGF-R2^+^ D-EPCs dysfunction observed through a negative effect of hyperglycemia-induced oxidative stress on the regulatory interaction between *Pik3c2a*/*Foxo1* and the possible activation of the *P66shc-Akt-Foxo* pathway. The fact that *Pik3c2a* is up-regulated in Lin^-^/VEGF-R2^+^ D-EPCs is supported by studies that demonstrated an inhibitory effect of up-regulated *Pik3c2a* on epithelial and cancer cell proliferation, migration and colony formation [40] illustrating the observed dysfunction of Lin^-^/VEGF-R2^+^ D-EPCs. Additionally, a recent study showed decreased expression of EPC *Pik3c2a* in coronary artery disease reducing their angiogenic and vasculogenic abilities [41], which highlights its importance in blood vessels repair that is impaired in diabetes.

*Ptf1a* is a pancreatic TF known for its role in pancreatic development and differentiation [42]. Lin^-^/VEGF-R2^+^ D-EPCs showed upregulation of *Ptf1a* that may be a compensatory action in diabetes. In parallel, the *Foxo4-Ptf1a* pathway plays a central role in directing the differentiation of retinal progenitor cells [43]. Thus, impaired *Foxo4-Ptf1a* pathway may cause diabetic retinopathy.

The implication of *Ahr* in diabetes or in EPC function has never been described before. *Ahr* is a cytosolic TF that when activated translocate to the nucleus altering the transcription of target genes. Here, *Ahr* was DE and its role in the D-EPC-GRN seems to be fundamental. It is one of the central-hubs, regulating *Clcnka* and *Ptf1a*, two of the three diabetic genes. Moreover, *Ahr* is also prominent in the 3-node co-regulatory FFL-motifs (Fig 4) comprising the DE TFs/genes and the miRNAs in the D-EPC-GRN where *Ahr* activates *Foxa2* and *Nfkb1*. *Mir-124* was also found to repress *Foxa2* and *Ahr* as well as *Sox9*, all of which important key genes in diabetes. Therefore, we believe that *Ahr* plays a significant role in controlling D-EPC.

*Clcnka* is a poorly studied gene that we found to be downregulated in Lin^-^/VEGF-R2^+^ D-EPCs. Mutations in this gene have been linked to dysfunctional endothelium, hypertension and cardio-renal axis dysfunction [44]. Therefore, we assume that diabetes influenced *Clcnka* downregulation contributes to the capacity of Lin^-^/VEGF-R2^+^ EPCs to regenerate endothelium in organs such as kidneys, which are damaged in diabetes. Chloride regulation is not really well understood but it has been related to the regulation of proinsulin conversion to active-insulin. *Clcn3*(-/-) mice demonstrate impaired insulin secretion demonstrating an important role of chloride channel proteins in insulin processing and secretion [45], which supports the implication of *Clcnka* in impaired function of D- Lin^-^/VEGF-R2^+^ EPCs especially via *Ahr* and *mir-124* as seen in Fig 4. It is also important to mention that hereditary diabetes insipidus patients demonstrated mutated *Clcnka* [46].

This study also identified 27 miRNAs that are associated with the regulation of the 80 DE diabetic-genes, which were statistically enriched with EPC-related biological processes such as cell proliferation, differentiation, cell-fate determination, hematopoiesis, glucose metabolism, and diabetes mellitus. Many of the 27 miRNAs were previously found to be associated with progenitor cells function and diabetes complications. For instance, *mir-451* level has been reported to be lower in hematopoietic progenitor cells as compared to controls [47]. In this study *mir-451* is DE in non-hematopoietic progenitor cells and is regulated by *Myc*, a chief regulatory gene in D-EPC-GRN. *Myc* activation is reported to regulate cell growth (upregulates rRNA), cell proliferation (upregulates cyclins, downregulates p21), differentiation, stem cell self-renewal, and apoptosis (downregulates Bcl-2) [47]. Forced expression of four mouse stem cell factors (*Oct4*, *Sox2*, *Klf4*, and *Myc*) changes the phenotype of rat endothelial cells to vascular progenitor cells [48]. Such vascular progenitor cells share cardinal properties with our Lin^-^/VEGF-R2^+^ EPCs samples where *Myc* was DE. This emphasizes the isolation efficiency of our Lin^-^/VEGF-R2^+^ EPCs. Additionally, *mir-223* is regulated by *Cebpa* through *Foxq1*. Since *mir-223* expression is usually high in essential thrombocythemia and primary myelofibrosis group [49], this may indicate the importance of this miRNA in stem cell differentiation into the myeloid lineage.

Importantly, we found an interesting relationship between *Cebpa* which regulate *Myc* and *mir-709* at the same time, which then represses *Myc* activation through a negative feedback mechanism. In parallel to this, *mir-709* suppresses the expression of three other genes, *Slc2a13*, *Lhx3* and *Esrra*, which are all activated by *Myc* underlining the central role of both *Myc* and *mir-709* in D-EPC function (Panels A and B in S3 Fig). Potential therapies can be directed against such genes and miRNAs to alter the fate of the Lin^-^/VEGF-R2^+^ D-EPCs and restore their function.

Interestingly, our study identified multiple miRNAs (*miR-1*, *miR-206*, *mir-133a*, *mir-103*, *mir-107*, and *mir-223*) that have been linked to diabetes. For example, in both *in vivo* and *in vitro* experiments, increased levels of glucose in myocardiomyctes led to significant upregulation of *miR-1* and *miR-206* with resulting modulation of *Hsp60* leading to accelerated glucose-mediated apoptosis in cardiomyocetes [50] and *mir-133a* was downregulated in diabetic cardiomyopathy [51]. Altered activation of *PI3K* and *SREBP-1c* may explain the defective regulation of *miR-1* and *miR-133a* expression in response to insulin in muscle of type-2 diabetic patients [52]. *Mir-103* and *mir-107* were noted to be upregulated in obese mice and were subsequently found to have a key role in insulin sensitivity making them potential targets for the treatment of type-2 diabetes [53]. Also *mir-223* was reported to be associated with type-2 diabetes [54].

Similarly, other miRNAs that were identified in this study (*mir-223*, *mir-205*, and *mir-210*) are linked with proliferation and differentiation of hematopoietic progenitor cells. Analyses of expression profiles indicated that *mir-223* expression decreases as cells mature during monocytic, erythroid, and mast cell differentiation. *mir-223* down-regulation during erythropoiesis is required for erythrocyte proliferation and differentiation at progenitor and precursor level [55]. In Lin^-^/VEGF-R2^+^ D-EPCs *mir-223* is suppressing *Mef2c*, which is activated by *Nkx2-2* (Fig 4). This is a novel relationship that has not been reported before. *Mir-205* plays a role in directing stem cell fate [56]. A study on mammary-gland progenitor cells showed that *miR-205* overexpression led to an expansion of the progenitor-cell population, decreased cell size and increased cellular proliferation [56]. In this study, *mir-210-3p* is able to suppress *Nfkb1* that is activated by *Ahr* and/or *Gata1* (Fig 4). *Mir-210* in particular, has been studied for its effects in rescuing cardiac function after myocardial infarcts via the upregulation of angiogenesis and inhibition of cardiomyocyte apoptosis [57].

There is strong evidence that supports the concept of diabetes altering the number of circulating Lin^-^/VEGF-R2^+^ EPCs (60), which are likely trapped in BM, and impairing their vasoreparative potential resulting in premature senescence (61). Thus, it is likely that diabetes influences the expression of genes in Lin^-^/VEGF-R2^+^ EPCs that are specific to those pathways. Although many studies reported that diabetes causes reduction in PB EPC number [5], others have reported an increase in EPC number in the circulation [58] while we did not find any significant effect of diabetes in mice [7]. We previously reported downregulation of *Sdf-1* and *Sele* genes in Lin^-^/VEGF-R2^+^ D-EPCs [31]. Since Lin^-^/VEGF-R2^+^ EPCs have the ability to produce SDF-1 [59] and SDF-1*/*Cxcr4 is a known EPC mobilization and maturation axis [60], this downregulation of *SDF-1* may contribute to the impaired mobilization of Lin^-^/VEGF-R2^+^ D-EPCs. Thus the observed decrease of Lin^-^/VEGF-R2^+^ D-EPCs in PB could be attributed to the impaired mobilization ability from BM to PB leading to EPC BM-trapping and not to the impaired proliferation. Based on the MGI database [14] *eNOS*, *Sdf-1*, *Cxcr4*, and *Sele* are all specific EPC genes but in this study they were not among the DE gene list. Despite that, we found that these genes are regulated by some of the central-hub genes and miRNAs in our D-EPC-GRN in particular *mir-139-5p*, *mir-709*, *Gata1*, *Ppara*, and *Tbp* (S4 Table). It should be mentioned that these regulatory interactions are based on predicted evidences through regNET database [61] rather than experimental evidence.

Dysfunction of *Nos3* signaling has also been implicated in EPC dysfunction in diabetes. The dysfunction has been linked with decreased *Nos3* activity [62] and the *Nos3* deficient (*NOS3*^-/-^) mouse had impaired EPC mobilization and angiogenesis [62]. The expression and phosphorylation of *Nos3* are essential for the survival, migration and angiogenesis facilitated by Lin^-^/VEGF-R2^+^ EPCs and ECs [63]. Human EPCs that overexpress *Nos3* have increased migratory potential, increased ability to incorporate into tube-like structures and to differentiate into endothelial spindle-like structures [64]. We did not observe a significant change in *Nos3* expression in Lin^-^/VEGF-R2^+^ D-EPCs. Based on the predicted analysis *Nos3* is regulated by *mir-139-5p*, *Gata1* and *Ppara*. We previously reported that *Nos3* expression in BM Lin^-^/VEGF-R2^+^ EPCs was very low indicating that they are early progenitor cells [31] since late EPCs have higher expression *Nos3* levels [65].

In conclusion we were able to detect associated genes, miRNAs, and collaborative network modules that are affected by early stages of diabetes in BM Lin^-^/VEGF-R2^+^ EPCs. To our knowledge, this is the first report that predicts and unravels the combinatorial regulatory interactions and genetic alterations that are specific to Lin^-^/VEGF-R2^+^ D-EPCs. Specific findings such as *Ppar* signaling pathway and *Foxo1&4-mic139-5p-Gata1-Pou3f1* axis and their role in Lin^-^/VEGF-R2^+^ EPC proliferation and differentiation and in insulin signaling pathway, *Pik3c2a-Ptf1a-Foxo4-Ahr* axis and its role in early onset of diabetes and blood vessel repair, *P66shc-Akt-Foxo* pathway and its role in oxidative stress associated diabetes and EPC dysfunction, as well as *Cebpa-Myc-mic-709* axis and its role in D-EPC function may lead to novel therapeutic strategies for mobilization of Lin^-^/VEGF-R2^+^ EPCs and the treatment of diabetic vascular complications such as diabetic retinopathy, nephropathy and cardiovascular disease.

## Supporting information

S1 Table List of all differentially expressed genes. There were 80 genes specific to comparison 4 (non-diabetic EPC vs D-EPC) and they were all annotated in the *Mouse WG-6 V2 beadchip*. There were only 3 genes that are diabetes related. They are underlined and *italic* formatted. Genes with positive LFC Diff values are up regulated in D-EPC.
(DOC)

S2 Table The annotated functional terms and diseases in the identified miRNA list. (DOC)

S3 Table The complete list of detected TF-miRNA co-regulatory motifs. (DOC)

S4 Table Predicted interactions between EPC specific functional DE-genes (Cxcr4, Nos3, Cxcl12) and genes in our D-EPC-GRN. (DOC)

S1 Fig Principal Component Analysis (PCA) for all identified 80 genes in comparison 4 (non-diabetic and diabetic EPCs). Genes are clustered based on relative gene expression and are given a color-coded sphere. Green spheres are genes that are downregulated. Red spheres are genes that are up-regulated.
(TIF)

S2 Fig The entire TF-miRNA network (D-EPC-GRN) constructed from the differentially expressed genes, their targets and regulators as well as the enriched miRNAs and their targets and regulators. Nodes in turquoise triangle denote TFs. The miRNAs are represented in orange square shapes. Grey circles represent the target genes. Larger nodes (forming the inner circle) are the identified central-hubs that might act as putative driver TFs/miRNAs. Black solid arrows indicate the regulation of TFs to target genes. Black dotted arrows indicate the regulations of TFs to miRNAs. The repression of miRNAs to their target genes is represented in red dotted arrows.
(TIF)

S3 Fig Panels A and B. Visualization of all motifs that contain mir-709 and its interactions with central-hubs and other genes.
(EPS)
