# Supplementary material for: Identification of novel diabetes impaired miRNA-transcription factor co-regulatory networks in bone marrow-derived Lin-/VEGF-R2+ endothelial progenitor cells
Source: PLoS One. 2018 Jul 11;13(7):e0200194. doi: 10.1371/journal.pone.0200194 (PMC6040716; doi:10.1371/journal.pone.0200194)
Supplement: S1 Table — There were 80 genes specific to comparison 4 (non-diabetic EPC vs D-EPC) and they were all annotated in the Mouse WG-6 V2 beadchip. There were only 3 genes that are diabetes related. They are underlined and italic formatted. Genes with positive LFC Diff values are up regulated in D-EPC. (DOC) [file pone.0200194.s001.doc]

**S1 Table.**

| **Symbol** | **Entrez Gene ID** | **LFC Diff** | **Regulation** |
| --- | --- | --- | --- |
| *0610035N01Rik* | NA | 6.09E-02 | UP |
| *1700060C20Rik* | NA | -5.22E-02 | DOWN |
| *1700061J05Rik* | 73376 | 8.27E-02 | UP |
| *2310011J03Rik* | 66374 | 1.48E-01 | UP |
| *2410018M08Rik* | 71970 | -7.78E-02 | DOWN |
| *3830431G21Rik* | 217682 | 6.19E-02 | UP |
| *4933405L10Rik* | 71046 | -5.62E-02 | DOWN |
| *A130095C03Rik* | NA | 6.69E-02 | UP |
| *A430108E01Rik* | NA | 7.13E-02 | UP |
| *AI987712* | NA | -4.26E-02 | DOWN |
| *Abcc2* | 12780 | -6.13E-02 | DOWN |
| *Ankrd50* | 99696 | 6.35E-02 | UP |
| *Ap2a2* | 11772 | 1.98E-01 | UP |
| *Aspscr1* | 68938 | 8.53E-02 | UP |
| *B530002L08* | NA | 5.29E-02 | UP |
| *B930026D14Rik* | NA | 5.22E-02 | UP |
| *BB146404* | 103511 | -4.81E-02 | DOWN |
| *Bccip* | NA | -1.40E-01 | DOWN |
| *Bmf* | NA | 2.60E-01 | UP |
| *Bxdc5* | 70285 | -2.03E-01 | DOWN |
| *Casp3* | NA | -7.24E-02 | DOWN |
| *Cbwd1* | 226043 | -1.15E-01 | DOWN |
| *Ccdc59* | 52713 | -1.14E-01 | DOWN |
| *Cct6b* | 12467 | 6.10E-02 | UP |
| *Cct7* | 12468 | -1.68E-01 | DOWN |
| *Cd33* | 12489 | 3.21E-01 | UP |
| *Cdyl* | NA | 2.87E-02 | UP |
| *Chmp4b* | 75608 | 1.61E-01 | UP |
| *Clcnka* | *12733* | *-7.02E-02* | *DOWN* |
| *Cngb1* | 333329 | -6.39E-02 | DOWN |
| *Colec11* | 71693 | 5.83E-02 | UP |
| *Cxx1c* | 72865 | -8.14E-02 | DOWN |
| *D030015B04Rik* | NA | -5.88E-02 | DOWN |
| *D230025D16Rik* | 234678 | -5.70E-02 | DOWN |
| *D430023I21Rik* | NA | 8.09E-02 | UP |
| *D430025H09Rik* | NA | 5.59E-02 | UP |
| *Ddx10* | 77591 | -1.06E-01 | DOWN |
| *Drd1a* | 13488 | 4.73E-02 | UP |
| *E430004N04Rik* | 210757 | -6.90E-02 | DOWN |
| *Fbxl17* | NA | 1.80E-02 | UP |
| *Fbxw12* | 434440 | -5.75E-02 | DOWN |
| *Gna11* | NA | 9.28E-02 | UP |
| *Iqcf4* | 67320 | -5.39E-02 | DOWN |
| *LOC100047905* | 100047905 | -2.55E-01 | DOWN |
| *LOC207678* | NA | 6.82E-02 | UP |
| *LOC208462* | NA | 5.46E-02 | UP |
| *LOC232364* | NA | -5.79E-02 | DOWN |
| *LOC245580* | NA | 8.19E-02 | UP |
| *LOC380897* | NA | -5.71E-02 | DOWN |
| *LOC382243* | 382243 | -6.54E-02 | DOWN |
| *LOC382866* | NA | 7.58E-02 | UP |
| *LOC382877* | NA | -5.92E-02 | DOWN |
| *LOC630980* | 630980 | -5.67E-02 | DOWN |
| *LOC637852* | 637852 | 4.65E-02 | UP |
| *LOC667776* | 667776 | -2.13E-01 | DOWN |
| *Mtap7d1* | 245877 | 2.16E-01 | UP |
| *OTTMUSG00000012631* | 383678 | 6.54E-02 | UP |
| *Obox2* | 246792 | -6.35E-02 | DOWN |
| *Olfm2* | 244723 | 5.36E-02 | UP |
| *Olfr1241* | 258447 | -4.28E-02 | DOWN |
| *Olfr339* | 258951 | 7.10E-02 | UP |
| *Olfr615* | 259084 | -4.72E-02 | DOWN |
| *Pi4kb* | 107650 | 9.18E-02 | UP |
| *Pik3c2a* | *NA* | *6.14E-02* | *UP* |
| *Pik3r5* | 320207 | 5.53E-02 | UP |
| *Ppp4r2* | 232314 | -9.99E-02 | DOWN |
| *Prss28* | 114661 | -6.75E-02 | DOWN |
| *Psmc2* | 19181 | -2.19E-01 | DOWN |
| *Ptf1a* | *NA* | *4.81E-02* | *UP* |
| *Slc12a1* | NA | 1.97E-02 | UP |
| *Slc16a4* | 229699 | -7.74E-02 | DOWN |
| *Slc2a13* | 239606 | -1.15E-02 | DOWN |
| *Slc6a19* | NA | 5.88E-02 | UP |
| *Tkt* | NA | 5.67E-02 | UP |
| *Tmem136* | 235300 | 4.74E-02 | UP |
| *Tyrp1* | 22178 | -4.29E-02 | DOWN |
| *Wipi2* | 74781 | 1.65E-01 | UP |
| *Xlr4b* | 27083 | -5.30E-02 | DOWN |
| *mKIAA0728* | NA | 6.10E-02 | UP |
| *scl0002357.1_64* | NA | -5.11E-02 | DOWN |
