# Supplementary material for: Identification of novel diabetes impaired miRNA-transcription factor co-regulatory networks in bone marrow-derived Lin-/VEGF-R2+ endothelial progenitor cells
Source: PLoS One. 2018 Jul 11;13(7):e0200194. doi: 10.1371/journal.pone.0200194 (PMC6040716; doi:10.1371/journal.pone.0200194)
Supplement: S2 Table — (DOC) [file pone.0200194.s002.doc]

**S2 Table.**

| **Category** | **Term** | **Count** | **P-value** |
| --- | --- | --- | --- |
| Cluster | hsa-mir-1 cluster | 2 | 0.00222496 |
| Cluster | hsa-mir-182 cluster | 3 | 0.0991 |
| Cluster | hsa-mir-200a cluster | 3 | 0.0991 |
| Family | mir-8 family | 4 | 0.0205 |
| Function | Apoptosis | 6 | 0.01364317 |
| Function | Cardiogenesis | 2 | 0.00647674 |
| Function | Cell cycle related | 12 | 0.0101 |
| Function | Cell differentiation | 4 | 0.00653509 |
| Function | Cell proliferation | 5 | 0.00791011 |
| Function | Epithelial-mesenchymal transition | 8 | 0.00030938 |
| Function | Hormones regulation | 8 | 0.00556873 |
| Function | Human embryonic stem cell (hESC) regulation | 11 | 0.000797 |
| Function | Inflammation | 8 | 0.00030938 |
| Function | Muscle development | 3 | 0.01260445 |
| Function | adipocyte differentiation | 4 | 0.03453751 |
| Function | carbohydrate metabolism | 4 | 0.00013413 |
| Function | cell death | 8 | 0.00251693 |
| Function | cell fate determination | 4 | 0.03041632 |
| Function | cell motility | 3 | 0.02523556 |
| Function | circadian clock | 3 | 0.00946872 |
| Function | circadian rhythm | 3 | 0.01260445 |
| Function | glucose metabolism | 2 | 0.02959805 |
| Function | heart development | 3 | 0.00304415 |
| Function | hematopoiesis | 8 | 0.035 |
| Function | miRNA tumor suppressors | 9 | 0.0165 |
| Disease | Adenocarcinoma | 11 | 0.00000119 |
| Disease | Adrenocortical Carcinoma | 8 | 0.00084084 |
| Disease | Arrhythmias- Cardiac | 2 | 0.00222496 |
| Disease | Atherosclerosis | 3 | 0.01260445 |
| Disease | Breast Neoplasms | 17 | 0.00000031 |
| Disease | Carcinoma | 7 | 0.00221828 |
| Disease | Carcinoma- Hepatocellular | 12 | 0.00561 |
| Disease | Carcinoma- Renal Cell | 5 | 0.00566198 |
| Disease | Carcinoma- Spindle Cell | 3 | 0.00038358 |
| Disease | Cardiomyopathy- Hypertrophic | 3 | 0.02523556 |
| Disease | Cholangiocarcinoma | 2 | 0.01256991 |
| Disease | Colonic Neoplasms | 7 | 0.0108564 |
| Disease | Diabetes Mellitus | 3 | 0.013760164 |
| Disease | Diabetes Mellitus- Type 3 | 2 | 0.010902638 |
| Disease | Distal Myopathies | 2 | 0.00647674 |
| Disease | Endometrial Neoplasms | 4 | 0.00045173 |
| Disease | Endometriosis | 7 | 0.0269 |
| Disease | Esophageal Neoplasms | 3 | 0.00684657 |
| Disease | Fatty Liver | 2 | 0.00647674 |
| Disease | Gastrointestinal Neoplasms | 3 | 0.03054283 |
| Disease | Glomerulonephritis- IGA | 3 | 0.0991 |
| Disease | Head and Neck Neoplasms | 5 | 0.01072435 |
| Disease | Heart Defects- Congenital | 2 | 0.02033103 |
| Disease | Hepatitis B | 3 | 0.00684657 |
| Disease | Hypertension | 2 | 0.02959805 |
| Disease | Hypertrophy | 3 | 0.04279821 |
| Disease | Laryngeal Carcinoma | 3 | 0.02047888 |
| Disease | Learned Helplessness | 7 | 0 |
| Disease | Liver Fibrosis | 2 | 0.00647674 |
| Disease | Liver Neoplasms | 4 | 0.00995648 |
| Disease | Lung Neoplasms | 13 | 0.00010043 |
| Disease | Melanoma | 14 | 0.00598 |
| Disease | Muscular Disorders- Atrophic | 7 | 0.00496813 |
| Disease | Musculoskeletal Abnormalities | 2 | 0.00647674 |
| Disease | Myelodysplastic Syndromes | 4 | 0.00045173 |
| Disease | Myocardial Infarction | 3 | 0.03054283 |
| Disease | Neoplasms | 13 | 0.0696 |
| Disease | Nephrosclerosis | 5 | 0.00000101 |
| Disease | Neurodegenerative Diseases | 2 | 0.04021961 |
| Disease | Osteoarthritis | 2 | 0.00647674 |
| Disease | Ovarian Neoplasms | 12 | 0.00561 |
| Disease | Pain | 3 | 0.00038358 |
| Disease | Pancreatic Neoplasms | 8 | 0.0019544 |
| Disease | Precursor Cell Lymphoblastic Leukemia-Lymphoma | 3 | 0.01627068 |
| Disease | Prostatic Neoplasms | 7 | 0.01597329 |
| Disease | Retinal Degeneration | 4 | 0.00025947 |
| Disease | Rhabdomyosarcoma | 2 | 0.02033103 |
| Disease | Sepsis | 3 | 0.001797 |
| Disease | Stomach Neoplasms | 8 | 0.00284296 |
| Disease | Stroke | 2 | 0.02959805 |
| Disease | Thyroid Neoplasms | 4 | 0.00400649 |
| Disease | Trophoblasts | 2 | 0.04021961 |
| Disease | Urinary Bladder Neoplasms | 6 | 0.00363263 |
| Disease | Urothelial Carcinoma | 2 | 0.00222496 |
| Disease | Uterine Cervical Neoplasms | 3 | 0.03054283 |
