# Supplementary material for: Identification of novel diabetes impaired miRNA-transcription factor co-regulatory networks in bone marrow-derived Lin-/VEGF-R2+ endothelial progenitor cells
Source: PLoS One. 2018 Jul 11;13(7):e0200194. doi: 10.1371/journal.pone.0200194 (PMC6040716; doi:10.1371/journal.pone.0200194)
Supplement: S3 Table — (DOC) [file pone.0200194.s003.doc]

**S3 Table.**

| Type | TF | miRNA | Target Gene | P-value |
| --- | --- | --- | --- | --- |
| TF-FFL | Cebpa | mmu-mir-709 | Myc | 0.0005 |
| TF-FFL | Foxd3 | mmu-mir-709 | Lhx3 | 0.0009 |
| TF-FFL | Foxo4 | mmu-mir-139-5p | Foxo1 | 0.0012 |
| TF-FFL | Gata1 | mmu-mir-139-5p | Zeb1 | 0.0022 |
| TF-FFL | Gata1 | mmu-mir-141 | Zeb1 | 0.0024 |
| TF-FFL | Gata1 | mmu-mir-709 | Esrra | 0.003 |
| TF-FFL | Max | mmu-mir-709 | Esrra | 0.0057 |
| TF-FFL | Max | mmu-mir-709 | Slc2a13 | 0.006 |
| TF-FFL | Max | mmu-mir-709 | Myc | 0.006 |
| TF-FFL | Meis1 | mmu-mir-709 | Esrra | 0.0061 |
| TF-FFL | Pou3f1 | mmu-mir-139-5p | Foxo1 | 0.007 |
| TF-FFL | Pparg | mmu-mir-210-3p | Nfkb1 | 0.0078 |
| TF-FFL | Pparg | mmu-mir-709 | Esrra | 0.0079 |
| miRNA-FFL | Ahr | mmu-mir-124 | Foxa2 | 0.01 |
| miRNA-FFL | Myc | mmu-mir-709 | Esrra | 0.0113 |
| miRNA-FFL | Myc | mmu-mir-709 | Slc2a13 | 0.0114 |
| miRNA-FFL | Myc | mmu-mir-709 | Lhx3 | 0.0127 |
| Coreg-FFL | Ahr | mmu-mir-124a | Foxa2 | 0.0139 |
| Coreg-FFL | Ahr | mmu-mir-210-3p | Nfkb1 | 0.0151 |
| Coreg-FFL | Ahr | mmu-mir-709 | Nrf1 | 0.0178 |
| Coreg-FFL | Ar | mmu-mir-139-5p | Foxo1 | 0.0181 |
| Coreg-FFL | Ar | mmu-mir-210-3p | Nfkb1 | 0.0187 |
| Coreg-FFL | Bach1 | mmu-mir-29c | Yy1 | 0.0188 |
| Coreg-FFL | Cebpa | mmu-let-7c | Myc | 0.0193 |
| Coreg-FFL | Cebpa | mmu-mir-139-5p | Foxo1 | 0.0207 |
| Coreg-FFL | Cebpa | mmu-mir-196 | Hoxc8 | 0.0207 |
| Coreg-FFL | Foxd3 | mmu-mir-124 | Sox9 | 0.0212 |
| Coreg-FFL | Foxd3 | mmu-mir-145 | Sox9 | 0.0214 |
| Coreg-FFL | Gata1 | mmu-mir-196 | Hoxc8 | 0.0245 |
| Coreg-FFL | Gata1 | mmu-mir-200a | Zeb1 | 0.0257 |
| Coreg-FFL | Gata1 | mmu-mir-200b | Zeb1 | 0.0277 |
| Coreg-FFL | Gata1 | mmu-mir-210-3p | Nfkb1 | 0.0278 |
| Coreg-FFL | Gata1 | mmu-mir-429 | Zeb1 | 0.0289 |
| Coreg-FFL | Hnf4a | mmu-mir-139-5p | Foxo1 | 0.0292 |
| Coreg-FFL | Maf | mmu-mir-124 | Ahr | 0.0317 |
| Coreg-FFL | Max | mmu-let-7c | Myc | 0.0323 |
| Coreg-FFL | Max | mmu-mir-29c | Yy1 | 0.033 |
| Coreg-FFL | Myc | mmu-mir-29c | Yy1 | 0.033 |
| Coreg-FFL | Nfkb1 | mmu-let-7c | Myc | 0.0338 |
| Coreg-FFL | Nfkb1 | mmu-mir-709 | Myc | 0.0355 |
| Coreg-FFL | Nkx2-2 | mmu-mir-223 | Mef2c | 0.0362 |
| Coreg-FFL | Nkx2-2 | mmu-mir-27b | Mef2c | 0.0365 |
| Coreg-FFL | Pou3f1 | mmu-mir-196 | Hoxc8 | 0.037 |
| Coreg-FFL | Ppara | mmu-mir-210-3p | Nfkb1 | 0.0384 |
| Coreg-FFL | Ppara | mmu-mir-709 | Esrra | 0.0398 |
| Coreg-FFL | Tbp | mmu-let-7c | Myc | 0.0406 |
| Coreg-FFL | Tbp | mmu-mir-29c | Yy1 | 0.0407 |
| Coreg-FFL | Tbp | mmu-mir-709 | Myc | 0.041 |
| Coreg-FFL | Tcf7 | mmu-let-7c | Myc | 0.042 |
| Coreg-FFL | Tcf7 | mmu-mir-709 | Myc | 0.0427 |
| Coreg-FFL | Trp53 | mmu-let-7c | Myc | 0.0437 |
| Coreg-FFL | Trp53 | mmu-mir-139-5p | Usf1 | 0.0438 |
| Coreg-FFL | Trp53 | mmu-mir-210-3p | Nfkb1 | 0.0452 |
| Coreg-FFL | Trp53 | mmu-mir-709 | Esrra | 0.0468 |
| Coreg-FFL | Trp53 | mmu-mir-709 | Myc | 0.0478 |
