# Supplementary material for: Identification of novel diabetes impaired miRNA-transcription factor co-regulatory networks in bone marrow-derived Lin-/VEGF-R2+ endothelial progenitor cells
Source: PLoS One. 2018 Jul 11;13(7):e0200194. doi: 10.1371/journal.pone.0200194 (PMC6040716; doi:10.1371/journal.pone.0200194)
Supplement: S4 Table — (DOC) [file pone.0200194.s004.doc]

**S4 Table.**

| **category** | **regulator** | **target** | **evidence** | **source** |
| --- | --- | --- | --- | --- |
| mirna-gene | mmu-mir-139-5p | Cxcr4 | Predicted | regNet |
| mirna-gene | mmu-mir-139-5p | Nos3 | Predicted | regNet |
| mirna-gene | mmu-mir-709 | Cxcl12 | Predicted | regNet |
| tf-gene | Gata1 | Nos3 | Predicted | regNet |
| tf-gene | Meis1 | Cxcr4 | Predicted | regNet |
| tf-gene | Ppara | Nos3 | Predicted | regNet |
| tf-gene | Ppara | Cxcl12 | Predicted | regNet |
| tf-gene | Tbp | Cxcr4 | Predicted | regNet |
| tf-gene | Ikzf1 | Nos3 | Predicted | regNet |
| tf-gene | Foxj2 | Cxcr4 | Predicted | regNet |
