# Supplementary figures and images for: Identification of novel diabetes impaired miRNA-transcription factor co-regulatory networks in bone marrow-derived Lin-/VEGF-R2+ endothelial progenitor cells
Source: PLoS One. 2018 Jul 11;13(7):e0200194. doi: 10.1371/journal.pone.0200194 (PMC6040716; doi:10.1371/journal.pone.0200194)

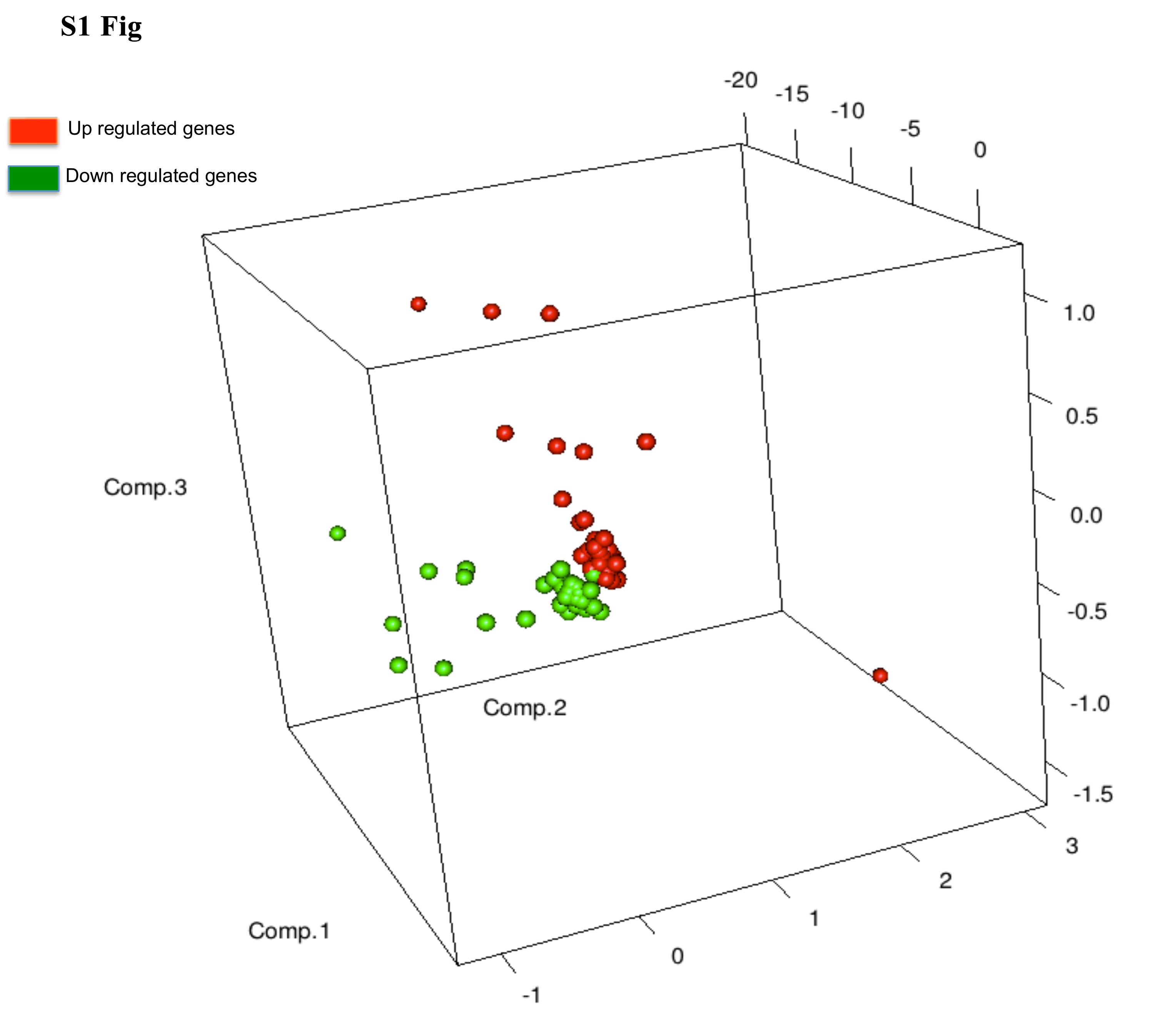

Supplement: S1 Fig — Genes are clustered based on relative gene expression and are given a color-coded sphere. Green spheres are genes that are downregulated. Red spheres are genes that are up-regulated. (TIF) [file pone.0200194.s005.tif]

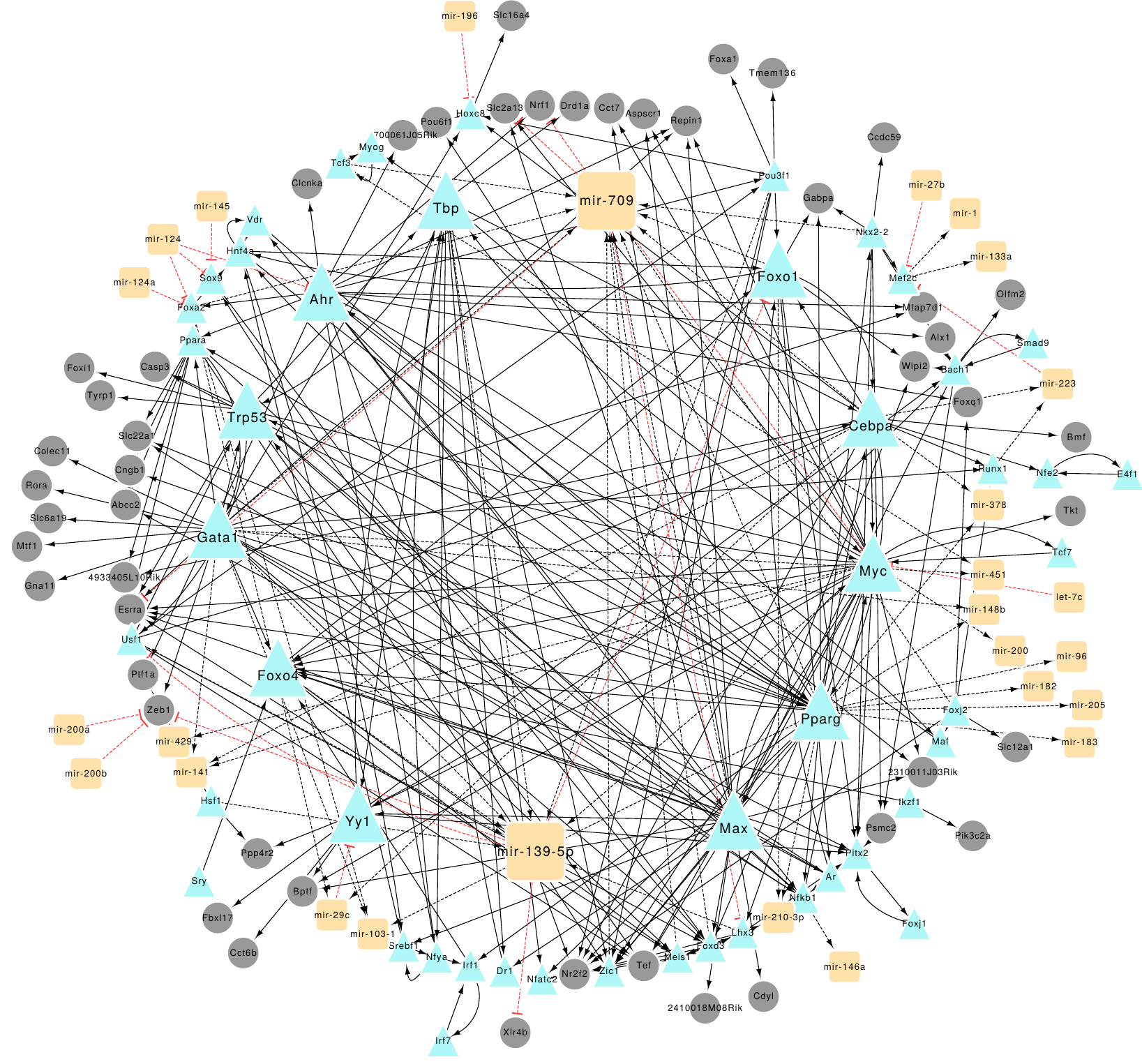

Supplement: S2 Fig — Nodes in turquoise triangle denote TFs. The miRNAs are represented in orange square shapes. Grey circles represent the target genes. Larger nodes (forming the inner circle) are the identified central-hubs that might act as putative driver TFs/miRNAs. Black solid arrows indicate the regulation of TFs to target genes. Black dotted arrows indicate the regulations of TFs to miRNAs. The repression of miRNAs to their target genes is represented in red dotted arrows. (TIF) [file pone.0200194.s006.tif]
